# The Hug of a Humpback Whale Mother: Protective Behaviors of a Calf Toward Escorts in a Competitive Group at Abrolhos Bank, Brazil

**DOI:** 10.3390/ani15243610

**Published:** 2025-12-15

**Authors:** Bianca Machado Righi, Laura Aguillar, Milton C. C. Marcondes, Yvonnick Le Pendu

**Affiliations:** 1 Instituto Baleia Jubarte, Caravelas 45900-000, Brazil; lalobastos.ppgzoo@uesc.br (L.A.); milton.marcondes@baleiajubarte.org.br (M.C.C.M.); 2 Laboratório em Mamíferos Aquáticos, Universidade Estadual de Santa Cruz, Ilhéus 45662-900, Brazil

**Keywords:** *Megaptera novaeangliae*, maternal care, harassment, drone, photogrammetry

## Abstract

**Simple Summary:**

We documented maternal protective behaviors in a female humpback whale in response to harassment from escort males within a competitive group. Video footage was obtained from aerial drone recordings on 3 August 2022, at the Abrolhos Bank, Brazil. We applied an aerial photogrammetry technique to estimate the size of the calf, the female, and the four harassing males. Based on drone video analysis, we describe for the first time a female’s protective behaviors toward her calf, using her body and pectoral fins during approaches by male escorts and physical contact between the escorts and the calf as they attempted to approach the lactating female. The use of drones allows us to describe new behaviors, even of well-known cetacean species.

**Abstract:**

While protective behaviors of baleen whales toward calves have been documented during predator attacks, they have not been observed in response to approach attempts by other males of the same species. We recorded high-resolution aerial drone videos of four competing male of humpback-whales (*Megaptera novaeangliae*) harassing a lactating female with her post-neonate calf in a breeding ground in Brazil. We document several protective behaviors and highlight a new role for pectoral fins in maternal protection, a function not previously attributed to this anatomical structure. These results confirm the value of using drones to describe new cetacean behaviors, even in well-known species.

## 1. Introduction

Protecting and nursing their young involves a significant energy expenditure for mysticetes [1,2] that undertake long migrations between feeding and breeding grounds. The lactating female humpback whale (*Megaptera novaeangliae*) fasts until she migrates back to her feeding grounds, relying solely on her energy reserves to maintain her own metabolism and ensure calf development [3]. During this period, postpartum ovulation can still attract males seeking to mate with these lactating females, which become potentially receptive [4]. When competing for access to a lactating female, males can negatively affect calf development by increasing travel time, decreasing rest periods [5], and disrupting nursing [6]. Furthermore, the aggressive behavior of males competing for access to the female increases the risk of physical injury and separation of the calf from its mother [5]. Therefore, describing how females avoid male harassment and protect their calves allows for an assessment of the additional energetic costs inherent to mother–calf interactions involving escorts during the early stages of calf development and maternal care.

In several populations, female humpback whales segregate into shallower areas to spatially distance themselves from competing males [6,7,8]. However, on the Abrolhos Bank, Brazil, the primary humpback whale breeding ground [9,10] has low bathymetric variation, which leaves mother–calf pairs closer and more accessible to escort males [11]. This makes it a good location for evaluating interactions between lactating females and harassing males. Protective behaviors toward calves have been described for several baleen whale species during predation attempts by killer whales (*Orcinus orca*) [12,13,14], but not in response to approach attempts by conspecific males. We describe novel behaviors performed by a lactating female humpback whale to protect her calf from harassment by competing males, as well as physical contact initiated by escorts against the calf during their attempts to approach the female.

## 2. Materials and Methods

The use of drones facilitates the identification of cetacean behaviors below the water surface and can triple the observation time compared to vessel-based researchers [15]. We used a DJI Phantom 4 PRO drone to apply the “focal group follow” [16,17] method at an altitude of 9.3 to 37 m and record high-resolution (4K) aerial videos of the mother–calf pair and the four escorts. Recordings were made with a drone-mounted camera with a gimbal that moved to allow observation of all individuals in the group. The lens was aimed vertically downward 90° to the horizon to extract measurements. The total length of each whale in the group was estimated using a vertical aerial photogrammetry technique based on calibrated images collected by the drone: an object of known length of one meter and a fixed flight height of 20 m were used as a scale reference to correct for any inaccuracies in the drone’s barometric altimeter. The size of the reference object was measured in pixels and converted to meters considering the altitude provided by the drone’s barometer, camera focal length, sensor size sensor, and image resolution [18]. Three measurements of each individual were taken and body length estimated using an adjusted R script [19] available at [20]. No image grading was carried out during analysis. To classify calves, we used their total length in relation to that of their mothers. Calves were considered neonates if they measured up to one third of the length of the mother, and post-neonates if they were longer [21]. We also used a threshold of >11.2 m to distinguish juvenile from adult [22]. Videos were transcribed into digitally tabulated behavioral data using the BORIS v. 8.21.8 video coding software [23]. We calculated the absolute frequency (number of occurrences) of two types of behavioral events: (1) evasive and protective behaviors of lactating females towards their calves, and (2) escorts’ harassing behaviors toward the female that resulted in physical contact with the calf.

## 3. Results

On 3 August 2022, we observed a group of humpback whales for 46 min from the research boat, consisting of a mother–calf pair accompanied by four escorts that were competing intensely for access to the female. The observation occurred in the Abrolhos Bank, Brazil (location: 18.05641° S, 38.79886° W), near the 27 m isobath. Although the sex of the escorts could not be determined from biopsy samples, we assumed they were all males based on their aggressive competitive behavior [5,24].

### 3.1. Photogrammetry and Photoidentification

The estimated total length was 12.1 ± 0.02 m for the adult female and 5.3 ± 0.14 m for the calf. Thus, the female, at over 11.2 m, was classified as an adult, and the calf, with a relative length of 43.8% of the mother’s total body length, was classified as a post-neonate. Escort 1 (12.9 ± 0.05 m), Escort 2 (11.2 ± 0.24 m) and Escort 3 (12.2 ± 0.09 m) were classified as adults, while Escort 4 (10.8 ± 0.03 m) was classified as a juvenile. Two individuals were photo-identified and added to the Happywhale platform, including Escort 1 (IBJ-7094) (https://happywhale.com/individual/93312, accessed on 28 August 2025) and the female (IBJ-7097) (https://happywhale.com/individual/93310, accessed on 28 August 2025). The female was sighted in a competitive group on the Abrolhos Bank in 2024 without a calf (IBJ data).

### 3.2. Video Analysis

We recorded a total of 25.15 min of video during the focal group follow. However, we analyzed 21.49 min of aerial video footage in which all group members were clearly visible in the frame. During approach attempts by the males, our analysis identified 42 instances of protective behavior by the female toward the calf and nine potentially dangerous surface events initiated by the escorts toward the calf (Table 1 and Videos S1–S6).

**Figure 1:**
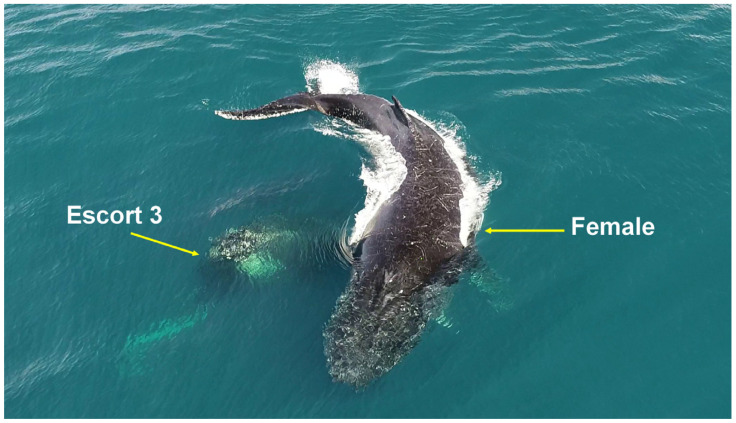
Acute reorientation behavior. A female humpback whale evading an escort with sharp turning angles on the Abrolhos Bank, Brazil.

During male approach attempts, the female altered her direction of travel with sharp turning angles (Figure 1) and exhibited behaviors toward the calf using her head and pectoral fins.

**Figure 2:**
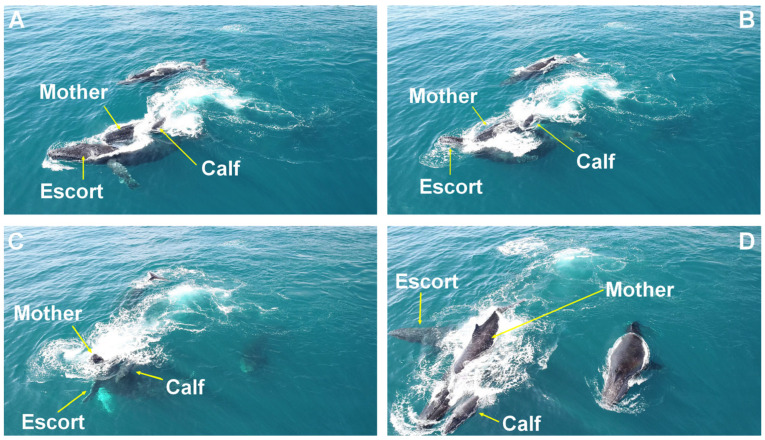
Head-push submersion behavior. Behavioral sequence of a female attempting to submerge a laterally approaching escort (**A**,**B**), creating an opening for the calf to move to the female’s opposite side (**C**,**D**). Observed on the Abrolhos Bank, Brazil.

**Figure 3:**
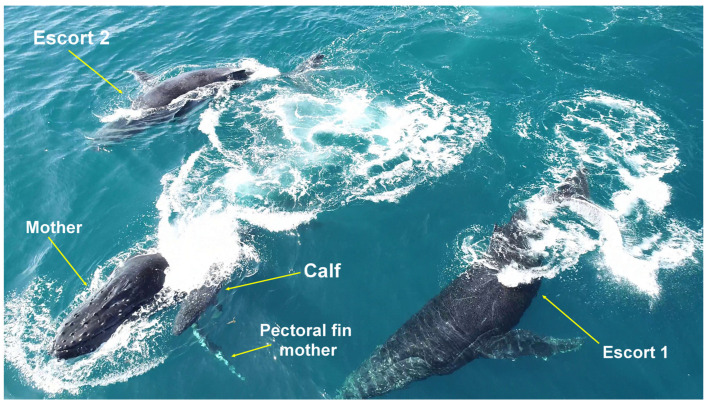
Pectoral fin carry behavior. A female moving with her calf on her left pectoral fin during an approach by escorts on the Abrolhos Bank, Brazil.

When an escort approached laterally, the female attempted to submerge it with her head, creating an opening for the calf to move to the opposite side of her body (Figure 2), or she moved with the calf positioned on either her right or left pectoral fin (Figure 3).

**Figure 4:**
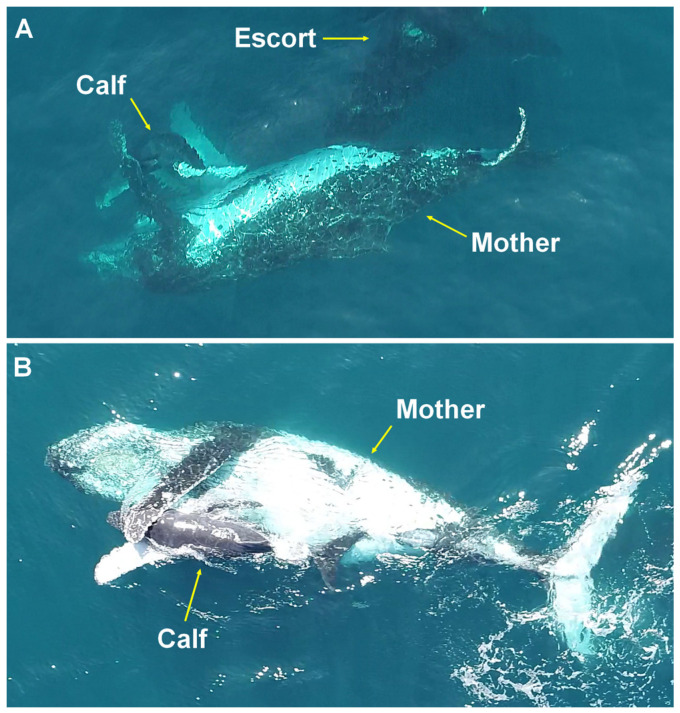
Hug behavior. A female diving with her young between her pectoral fins (**A**) and with the ventral side facing upward and crossing her pectoral fins around her calf (**B**), prior to an approach from below by escort males on the Abrolhos Bank, Brazil.

At other times, when one or more escorts approached from below or behind, the female drew the calf to her ventrum and extended her pectoral fins around it, sometimes crossing them (Figure 4).

The lactating female performed other behaviors already described in the literature, such as head and caudal fin slaps, to ward off the males and protect her calf (Table S1).

**Figure 5:**
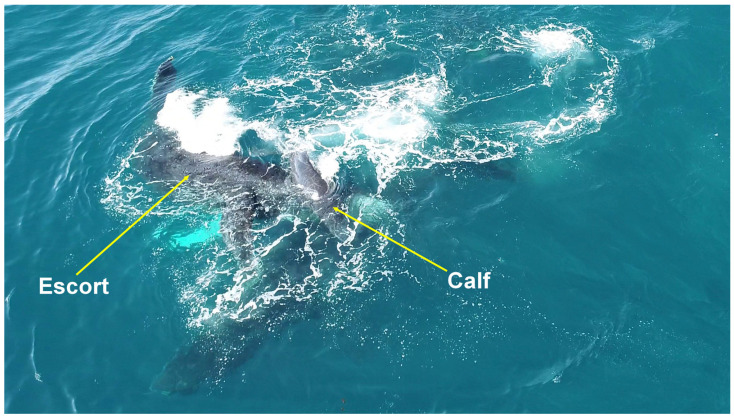
Head calf lift behavior. An escort lifts the calf with its head during an approach toward the female on the Abrolhos Bank, Brazil.

**Figure 6:**
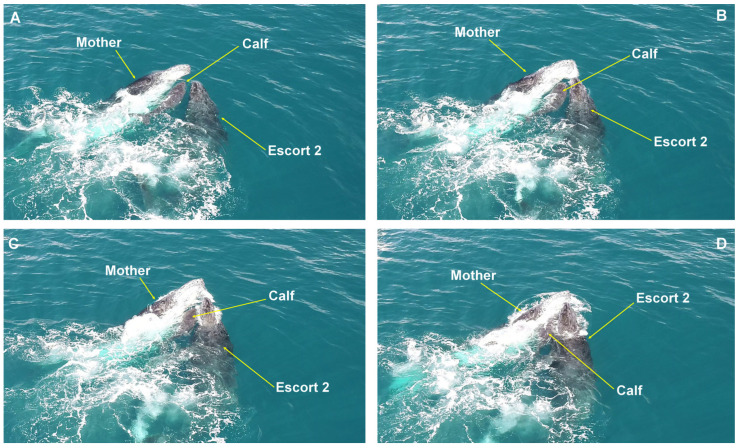
Calf compression behavior. (**A**–**D**) showing a lateral approach by an escort, who presses the calf between himself and the female. From a competitive group on the Abrolhos Bank, Brazil.

Despite the active protection of the female, escorts still managed to lift the calf with their heads on five occasions (Figure 5) and press the calf against its mother four times (Figure 6). Several behaviors characteristic of male–male competition were also recorded, such as “caudal fin slaps”, “extensions of the pectoral fin”, and “bubble trailing” [24] (Table S1).

## 4. Discussion

Humpback whale mother–calf pairs have been observed to strategically segregate into shallower waters to avoid male harassment in various breeding grounds, such as those in Ecuador [6,7] and Hawaiʻi [6,7]. In Serra Grande in Brazil, groups with calves were sighted in waters ten meters shallower than groups without calves [25]. However, the low depth variation in the Abrolhos Bank means that mother–calf pairs remain in close proximity and accessible to escorts [11]. In this context, a broad and complex repertoire of protective behaviors may supplement the habitat segregation strategy between males and females and be crucial for calf survival.

Acute reorientation was the most frequent behavior of the female to evade male harassment. In Hawaiʻi, calves exhibit significantly higher reorientation rates when in groups with multiple male escorts [5]. In Brazil, in a different context, a Bryde’s whale (*Balaenoptera edeni*) was observed frequently reorienting its swimming direction when near the potential danger posed by a vessel [26]. Thus, reorientation may be a behavior performed by female humpback whales in response to the threat posed by approaching males. However, these evasions from escorts can increase the risk of mother–calf separation, which poses a potential threat to the dependent calf.

The female repeatedly positioned her body to form a physical barrier between the calf and the males. Several cetacean species display similar protective behaviors during predation attempts by killer whales (*Orcinus orca*). Female gray whales (*Eschrichtius robustus*) position themselves between their calf and predators, in addition to striking vigorously with their tails [12]. In similar situations, humpback mothers keep their calf close while exhibiting aggressive behaviors such as tail slaps and surface lunges directed at the orcas [14]. Odontocetes exhibit similar behaviors: traveling spinner dolphin (*Stenella longirostris*) mothers protect their calves by positioning them between themselves and another female [27], while female Amazon river dolphins (*Inia geoffrensis*) exhibit the same behavior during infanticide attempts [28]. In response to harassment from escort males, the female humpback whale brought the calf close to her body and used a pectoral fin to hold it on top of them while she moved. The use of pectoral fin in maintaining interindividual relationships has been described as a strategy to fight off predatory attacks by killer whales in humpback whales, southern right whales (*Eubalaena australis*) [13], and dolphin species [29]. However, we believe our record of the humpback mother’s pectoral fin use is related to calf protection, as the “Pectoral Fin Carry” and “Hug” behaviors create a safe space/barrier against lateral and underneath approaches from escorts. One variation of the “Hug” behavior involved the female protecting her calf by holding it against her ventrum at the surface, a behavior previously described in female southern right whales during the breeding season in Brazil [30]. In a different context, during predatory attacks by killer whales, gray whales have also attempted to protect their calves by holding them on top of their ventrum [13]. Ventral exposure at the surface has been reported in adult humpback whales when attacked by orcas, but not for the purpose of protecting a calf [13]. This pectoral fin protection of a calf may be unique to humpback whales due to their long pectoral fins, which can reach one-third of the individual’s total length [31].

Active behaviors, such as head and tail slaps, corroborate the intense energetic investment of escort males competing for the female [24,32]. Despite the mother’s protective strategies, she could not always ensure the complete protection of her calf. Infanticide attempts, aimed at inducing copulation with the postpartum female, have been observed in bottlenose dolphins (*Tursiops truncatus*) [33], Indo-Pacific humpback dolphins (*Sousa chinensis*) [34], Guiana dolphins (*Sotalia guianensis*) [35], and killer whales [36]. Although never documented in baleen whales, the “head calf lift” and “calf compression” behaviors we describe do not appear to be infanticide attempts, as the primary objective of the escort males is to approach the female, not to sever the mother–calf bond or eliminate the offspring.

The presence of male escorts in groups of lactating females imposes significant energy costs on humpback whale mother–calf pairs. Females with calves are more likely to be pursued by multiple males than females without calves [37]. As a result, these female–calf pairs spend significantly more time on the move and less time resting when accompanied by escorts, compared to groups without male escorts [5,37]. For growing calves, an average reduction of 10% in surface time is due to the presence of escorts [5] and can lead to an increase in metabolic cost, which impacts on the energy allocated to their development.

In response to this pressure, lactating females may adopt counterstrategies to mitigate harassment from competitive males. One such strategy is to allow association with a single accompanying male [5,37], a behavior that aligns with the bodyguard hypothesis [38]. Although a recent study suggested that the association of females with humpback whale calves was related to the social development of the calves [39], it is also plausible that aggregations of females with calves function as a strategy to dilute the mother’s individual vigilance [40] toward approaching males. This could increase the time available for rest and potentially optimize energy allocation for lactation and calf growth.

The protective behaviors exhibited by the mother reveal a novel function for the pectoral fins of humpback whales, with implications for maternal care strategies, and consequently calf survival. Nevertheless, the behavior of competing males demonstrates plasticity in their strategies for approaching reproductive females and highlights gaps in our understanding of the social dynamics of humpback whale reproduction. Finally, we emphasize that the mother’s protective behaviors using her pectoral fins were not clearly identifiable to the naked eye by vessel-based researchers, as these actions occurred while the whales were submerged. This highlights the importance of using drones for studying cetacean behavior, as this aerial perspective allows for the identification of behaviors not visible from the horizontal viewpoint of vessel-based observers [41,42].

## 5. Conclusions

We identified several maternal protective behaviors involving the pectoral fins of humpback whales, a previously undocumented function for this organ. The large size of their pectoral fins is a unique characteristic among cetaceans, and the use of these appendages for protection may be exclusive to the species. Describing novel behaviors in one of the most studied cetacean species reinforces the value of drones as a research tool, providing a unique perspective that allows for detailed descriptions of social interactions.

## Figures and Tables

**Table 1:** Absolute frequency of novel humpback whale surface behaviors on the Abrolhos Bank, recorded during 21.49 min of aerial drone video. Behaviors include (a) male avoidance and calf protection by a lactating female, and (b) harassment of the female by four males, resulting in contact with the calf.

Behavioral Event	Description	Frequency
(a) Evasive and protective behaviors of lactating females towards their calves		
Acute Reorientation	A change in the direction of travel at an angle of less than 90°	18
Head-push Submersion	Pushing an escort underwater with her head, allowing the calf to move to her opposite side	5
Pectoral Fin Carry	The mother swimming with the calf positioned on one of her pectoral fins	8
Hug	Positioning and holding the calf between the pectoral fins, either with the mother’s ventrum facing up or down, and with the pectoral fins either crossed or uncrossed	11
(b) Escorts’ harassing behaviors toward the female that resulted in physical contact with the calf		
Head Calf Lift	Lifting the calf with the head	5
Calf compression	An escort approaches the female, squeezing the calf against the mother with its head or flank	4

## Data Availability

The original contributions presented in this study are included in the article/Supplementary Materials. Further inquiries can be directed to the corresponding authors.

## Supplementary Materials

The following supporting information can be downloaded at: https://www.mdpi.com/article/10.3390/ani15243610/s1, Table S1. present the frequency of behaviors performed by calf, female and escorts. Further supporting information can be downloaded at: https://www.ebi.ac.uk/biostudies/studies/S-BSST2101 (accessed on 28 August 2025). We present High resolution videos captured by drones to elucidate and facilitate understanding of the behaviors exhibited by the escort males and the mother–calf pairs. Video S1: Acute reorientation—A female humpback whale evading an escort with sharp turning angles. Video S2: Head-push submersion—A female pushing an escort underwater with her head, allowing her calf to move to her opposite side. Video S3: Pectoral fin carry—A female moving with her calf on her pectoral fin during an approach by escorts. Video S4: Hug—A female crossing her pectoral fins around her calf. Video S5: Head calf lift—An escort lifting the calf with its head during an approach toward the female. Video S6: Calf compression—An escort pressing the calf between himself and the female. Reference [24] is cited in the Supplementary Materials.
