# Supplementary material for: The Hug of a Humpback Whale Mother: Protective Behaviors of a Calf Toward Escorts in a Competitive Group at Abrolhos Bank, Brazil
Source: Animals (Basel). 2025 Dec 15;15(24):3610. doi: 10.3390/ani15243610 (PMC12729933; doi:10.3390/ani15243610)
Supplement: Supplementary file 1 [file animals-15-03610-s001.zip › animals-3783437-supplementary.pdf]

Supporting Information

Table S1. Frequency of behaviors performed by calf, female and escorts according to Baker, C.S.; Herman, L.M. 1984 [24].

**Individual**	**Behavioral event**	**Frequency**
Calf	Extensions of the pectoral fin	3
	Caudal fin slap	6
Female	Head slap	4
	Caudal fin slap	3
	Extensions of the pectoral fin	8
	Bubble trailing	2
Escorts	Head slap	5
	Caudal fin slaps	12
	Extensions of the pectoral fin	46
	Bubble trailing	15
